# The Effect of Shavings from 3D-Printed Patient-Specific Cutting Guide Materials During Jaw Resection on Bone Healing

**DOI:** 10.3390/ma18245624

**Published:** 2025-12-15

**Authors:** Erina Tsunoda, Masako Fujioka-Kobayashi, Masateru Koyanagi, Yuichiro Arai, Toru Inomata, Ryo Inada, Takafumi Satomi

**Affiliations:** 1 Department of Oral and Maxillofacial Surgery, School of Life Dentistry at Tokyo, The Nippon Dental University, Tokyo 102-8159, Japan; e-tsunoda@tky.ndu.ac.jp (E.T.); m-koyanagi0102@tky.ndu.ac.jp (M.K.); y-arai@tky.ndu.ac.jp (Y.A.); t-ino@tky.ndu.ac.jp (T.I.); iryoda@tky.ndu.ac.jp (R.I.); tsatomi@tky.ndu.ac.jp (T.S.); 2 Department of Oral and Maxillofacial Surgery, Shimane University Faculty of Medicine, Izumo 693-8501, Japan; 3 Department of Oral and Maxillofacial Surgery, Machida Municipal Hospital, Tokyo 194-0023, Japan

**Keywords:** cutting guide, biocompatibility, jaw resection, 3D printer

## Abstract

Patient-specific cutting guides are used for safe and accurate jaw resection during oral and maxillofacial surgery. This study investigated the effect of shavings from 3D-printed cutting guide materials during surgery on bone healing. The biocompatibility of commercially available biocompatible polymers including photopolymer resin (PP) and polyamide resin (PA) materials was assessed in the present study. The viability of mouse osteoblast-like MC3T3E-1 cells was evaluated upon coculture with the materials. Furthermore, the effects of PP and PA as additives on bone formation were investigated in a rat calvarial bone defect model. Both PP and PA were biocompatible and allowed cells to attach to them. However, both materials could be damaged when cutting devices were used, and their shavings impaired osteoblast proliferation and bone formation. Cutting guide materials are designed to be biocompatible when they are fabricated according to the manufacturer’s protocol. Nevertheless, the polymer shavings generated during jaw cutting might adversely affect bone healing.

## 1. Introduction

Advances in digital technologies, including imaging software, computer-aided design/manufacturing (CAD/CAM), and additive manufacturing, have transformed three-dimensional (3D) object fabrication and are being increasingly applied in clinical practice [1,2] In oral and maxillofacial surgery, computer-aided surgery (CAS) has enabled CT-based virtual planning and guided procedures, particularly in mandibular reconstruction following tumor resection [3,4,5]. The precise location of osteotomy for marginal or segmental mandibulectomy can be determined by 3D-printed cutting guides, which greatly improve efficiency and accuracy [6].

Vat photopolymerization (VPP) technology is used to create 3D objects by selectively curing liquid photopolymers through light-activated polymerization [7]. Various synthetic polymers have been used for maxillary and mandibular cutting guides, including photopolymer resins (PP), polyamides (PA), thermoplastic polyurethanes, and acrylic resins [8]. Both PP and PA materials offer strength, biocompatibility, and high precision and detail, making them popular choices for cutting guides [4,8,9]. This study focused on PP materials fabricated by VPP technology and stereolithography (SLA) 3D printing, and PA materials fabricated by powder bed fusion (PBF) technology and selective laser sintering (SLS), since these are materials commonly used for cutting guides during jaw resection in oral and maxillofacial surgery.

Among the numerous reports of surgical guides fabricated from PP and PA, most have focused on the dimensional accuracy and clinical utility, with few focusing on their biocompatibility [10,11,12], since commercially available PP and PA materials are considered safe and biocompatible on the basis of their ISO 10993 test results before clinical application [13,14]. However, the detached particulate debris/shavings from the cutting guides during surgery could enter the surgical field during intraoperative manipulation, and the shavings from PP and PA might influence the biocompatibility and bone healing capacity after bone reconstruction surgery. Nevertheless, the effects of the shavings from the cutting guides on biological tissues remain poorly understood.

It was hypothesized that the shavings from PP and PA might adversely affect bone healing even if the materials themselves are considered biocompatible on the basis of the standard. Therefore, the aim of the present study was to investigate the biological effects of shavings of 3D-printed cutting guides fabricated from PP and PA. The particle morphologies of both materials were examined using scanning electron microscopy (SEM), their effects on cellular responses were evaluated in vitro. Furthermore, a rat calvarial defect model was used for in vivo studies to assess how these materials influence the bone healing process in bone defects.

## 2. Materials and Methods

### 2.1. Preparation of Cutting Guide Materials

Photopolymer resin (PP) and polyamide resin (PA) were employed in the present study. The PP samples were fabricated in-house with a 3D printer (Form3B^®^, Formlabs, Somerville, MA, USA) and 3D-printing biocompatible resin (Surgical Guide Resin^®^, Formlabs) according to the manufacturer’s protocol. The PP materials were composed of methacrylate monomers, urethane dimethacrylate and photoinitiators. The PA samples were obtained from patient-specific cutting guides of the TruMatch Reconstruction System^®^ provided by DePuy Synthes (Warsaw, IN, USA). Since the material composition is proprietary, except for the polyamide component, the unused parts of the cutting guides, which had been primarily ordered for patients, were utilized in the present study. This study was approved by the ethics committee of Nippon Dental University, Tokyo, Japan (approval number; NDU-T2024-19).

### 2.2. Preparation of the Material Shavings

The surfaces of both PP and PA were abraded using two different devices, a reciprocating saw (TPX/Core2^®^, Stryker Corporation, Kalamazoo, MI, USA) and an ultrasonic surgical unit (VarioSurg3^®^, NSK, Tochigi, Japan), each for 60 s of continuous cutting. The damaged surfaces of the materials were observed by scanning electron microscopy (SEM). The shavings of the materials were prepared and collected for the biocompatibility tests. Morphological observation of the material shavings was performed using a stereomicroscope (Axio Zoom V16, Carl Zeiss, Jena, Germany) and SEM.

### 2.3. SEM Imaging

The samples were mounted on aluminum stubs with carbon tape (E-1030 Ion Sputter, Hitachi High-Tech, Tokyo, Japan) and coated with osmium. SEM images were acquired using a scanning electron microscope (JSM-IT200, JEOL Ltd., Tokyo, Japan).

### 2.4. Cell Culture

Mouse osteoblast-like MC3T3-E1 cells (RCB1126, RIKEN, Saitama, Japan) [15] were maintained in α-MEM (Gibco, Life Technologies, Carlsbad, CA, USA) supplemented with 10% fetal bovine serum (FBS; Gibco) and 1% antibiotics (Anti-Anti, Gibco) at 37 °C in a humidified atmosphere containing 5% CO_2_. PP and PA disks were prepared with a diameter of 12 mm and a thickness of 2 mm. MC3T3-E1 cells were seeded on three types of disks at a density of 1.0 × 10^4^ cells per well in a 24-well plate (CELLSTAR^®^, Greiner Bio-One, Kremsmünster, Austria): (1) a Control plastic culture cover slip (Cell Desk LF, Sumitomo Bakelite, Tokyo, Japan) [16], (2) a PP disk, and (3) a PA disk. Twenty-four hours after seeding, the cells cultured on the disks were fixed overnight at 4 °C in 2.5% glutaraldehyde solution (FUJIFILM Wako Pure Chemical Corp., Osaka, Japan), dehydrated in a graded ethanol series and dried with hexamethyldisilazane (Sigma-Aldrich, St. Louis, MO, USA) for SEM observation.

### 2.5. Cell Proliferation Assay

Eluates were obtained from PP and PA shavings immersed in α-MEM supplemented with 1% antibiotics at a concentration of 5 mg/mL or 50 mg/mL after 24 h of incubation and stored at −20 °C. Cells were cultured in basal growth medium supplemented with 20% eluate. Cell proliferation assays were performed 1, 3 and 5 days after seeding using an MTS colorimetric kit (Promega, Madison, WI, USA), and the absorbance at 490 nm was measured with a microplate reader (SH-9000, CORONA ELECTRIC, Ibaraki, Japan).

### 2.6. Animals

Twenty male Jcl: Wistar rats (9 weeks old, 250–300 g; CLEA Japan, Inc., Tokyo, Japan) were used in this study. The animal study was approved by the Animal Care and Use Committee of the Nippon Dental University, School of Life Dentistry in Tokyo, Tokyo, Japan (Approval No. 24-13-2). The animals were housed individually (one rat per cage) without excessive or disruptive noise and fed a standard diet and water ad libitum in the Central Animal Care Facility at the Nippon Dental University.

### 2.7. Calvarial Bone Defect Assay

General anesthesia was induced via intraperitoneal injection of a mixed solution containing butorphanol tartrate (2.5 mg/kg; Meiji Seika Pharma Co., Ltd., Tokyo, Japan), medetomidine hydrochloride (0.15 mg/kg; Nippon Zenyaku Kogyo Co., Ltd., Fukushima, Japan), and midazolam (2 mg/kg; Astellas Pharma Inc., Tokyo, Japan). The cranial region was shaved, disinfected with 70% ethanol, and locally anesthetized with 1.0 mL of 2% lidocaine (AstraZeneca, Osaka, Japan). A midline skin incision of approximately 2 cm was made, and the periosteum was elevated to expose the calvarium. Two symmetrical circular bone defects (each 5 mm in diameter) were created in the parietal bone using a trephine bur under continuous sterile saline irrigation, taking care to avoid injury to the dura mater. Autograft bone material was prepared by crushing the drilled bone fragments with a rongeur. The defects were randomly assigned to one of four groups: (1) defect only (Control), (2) 22.5 mg of autograft (Bone), (3) 15.0 mg of autograft + 7.5 mg of PP shavings (Bone + PP), and (4) 15.0 mg of autograft + 7.5 mg of PA shavings (Bone + PA) using a computer-generated randomisation sequence created in Microsoft Excel. A minimal sample size of five animals per experiment was estimated by G*power [17]. Each defect was covered with a collagen barrier membrane (BioGide^®^, Geistlich Pharma AG, Wolhusen, Switzerland), and closure was performed by suturing the periosteum with 4-0 Vicryl^®^ (Ethicon, Somerville, NJ, USA) and the skin with 5-0 nylon sutures (Akiyama Medical MFG, Tokyo, Japan). At 2 and 8 weeks post-surgery, the animals were euthanized by intraperitoneal administration of pentobarbital sodium at a dose of 200 mg/kg.

### 2.8. Micro-CT Analysis

Calvarial specimens were fixed in 10% neutral buffered formalin (FUJIFILM Wako Pure Chemical Corp.) for 3 days, followed by transfer to 70% ethanol. Micro-CT images were acquired with a Scan Xmate-D100SS270 (Comscan, Kanagawa, Japan) at a voxel size of 31.847 µm. Image analysis was conducted with Amira 3D software (Thermo Fisher Scientific, Waltham, MA, USA). A cylindrical volume of interest (VOI; 5 mm diameter) corresponding to the defect was defined, in which the new bone area (NBA, %), new bone volume (NBV, mm^3^), and bone mineral density (BMD, mg/cm^3^) were measured.

### 2.9. Histomorphometric Analysis

The samples were dehydrated through a graded ethanol series and embedded in methyl methacrylate (Tokyo Chemical Industry Co., Ltd., Tokyo, Japan). Tissue blocks were cut through the center of the defect to obtain approximately 1000-µm-thick ground sections using a low-speed diamond saw (ISOMET, BUEHLER, Lake Bluff, IL, USA). The sections were mounted on acrylic plates, polished to a final thickness of 250 µm, and subsequently stained with toluidine blue and basic fuchsin (Sigma-Aldrich). Images were obtained at low magnification with an AXIO Zoom V16 microscope and at high magnification with an AXIO Imager M2 microscope (Carl Zeiss). Histomorphometric analysis was performed on the defect area (5 mm diameter) defined as the region of interest (ROI). Using Photoshop CC (Adobe Systems, San Jose, CA, USA), the following parameters were quantified: new bone area (NBA, mm^2^), bone marrow area (BMA, mm^2^), soft/connective tissue area (STA, mm^2^), and horizontal defect closure (HDC, %).

### 2.10. Statistical Analysis

For the in vitro cell experiments, at least three independent samples were assessed, and the mean and standard error are presented. The cell proliferation assay results were analyzed using two-way analysis of variance (ANOVA) followed by Tukey’s post hoc test with GraphPad Prism version 9.0 (GraphPad Software, La Jolla, CA, USA). Other data were analyzed using one-way ANOVA with Tukey’s post hoc test. Statistical significance was set at *p* < 0.05.

## 3. Results

### 3.1. In Vitro Assessment of the 3D-Printed Cutting Guide Materials

**Figure 1:**
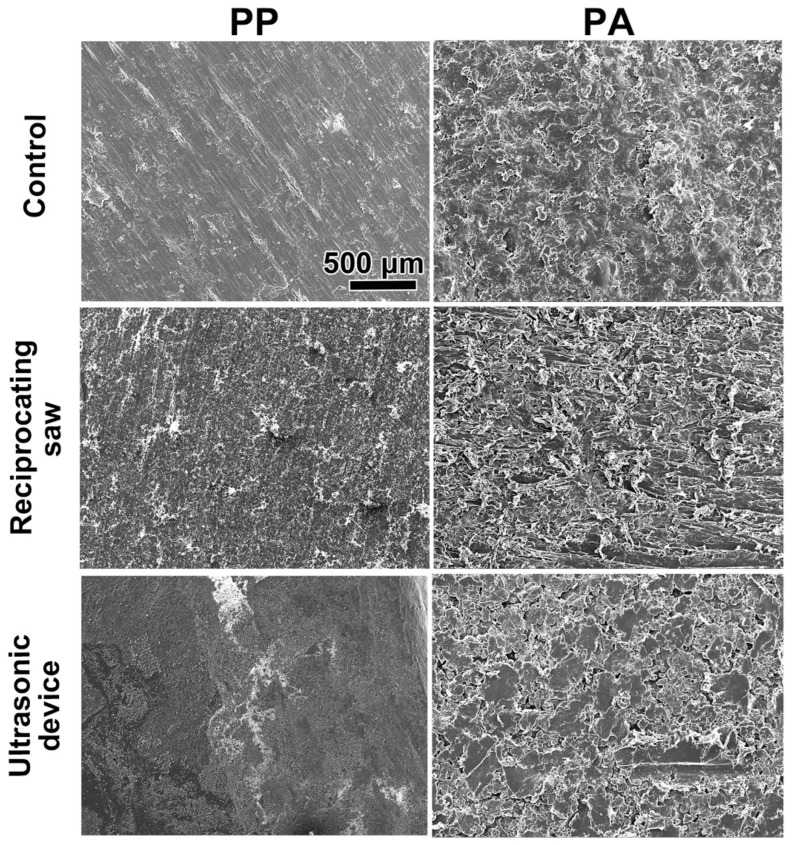
SEM images of the surfaces of the PP and PA damaged with two different cutting devices, a reciprocating saw and an ultrasonic device. Control represents undamaged samples. Scale bar applies to all images.

The 3D-printed cutting guides were analyzed to assess their material properties, cell viability and proliferation in vitro. First, the effects of cutting with the two different cutting devices—a reciprocating saw and an ultrasonic device—on the material surface were experimentally investigated and observed by scanning electron microscopy (SEM). The surfaces of the photopolymer resin (PP) and polyamide resin (PA) were damaged with both cutting devices (Figure 1). The intact surface of the PP was smooth and flat, whereas that of the PA was smooth with minor surface undulations. Compared with the ultrasonic device, the reciprocating saw created a rougher surface on both the PP and PA materials (Figure 1).

**Figure 2:**
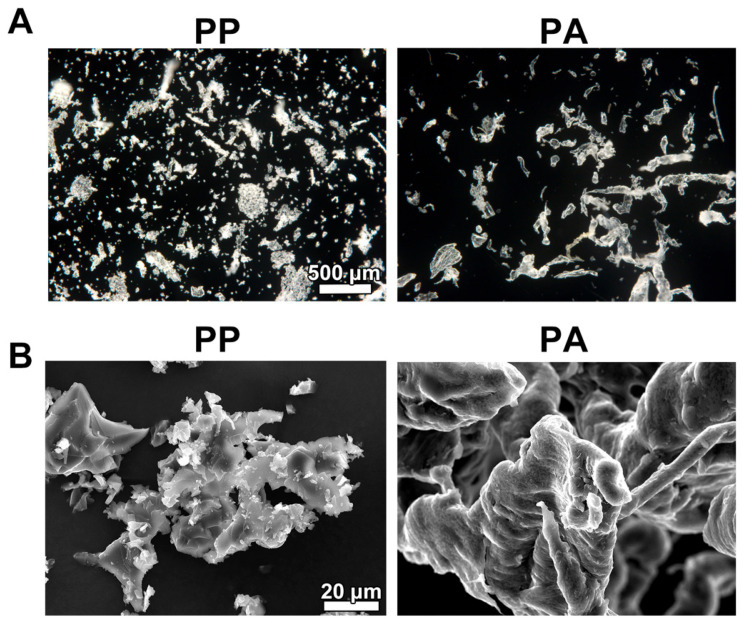
Images of the PP and PA shavings. (**A**) Stereomicroscopy images of the (1) PP and (2) PA shavings. Scale bar applies to both PP and PA images. (**B**) SEM images of the (1) PP and (2) PA shavings. Scale bar applies to both PP and PA images.

The shavings from the PP and PA materials were observed by stereomicroscopy and SEM (Figure 2), revealing crystal-like particles in the PP samples and short fibers in the PA samples (Figure 2A). SEM revealed that the PP particles had sharper edges with small particles, while the PA sample surfaces were smoother (Figure 2B).

**Figure 3:**
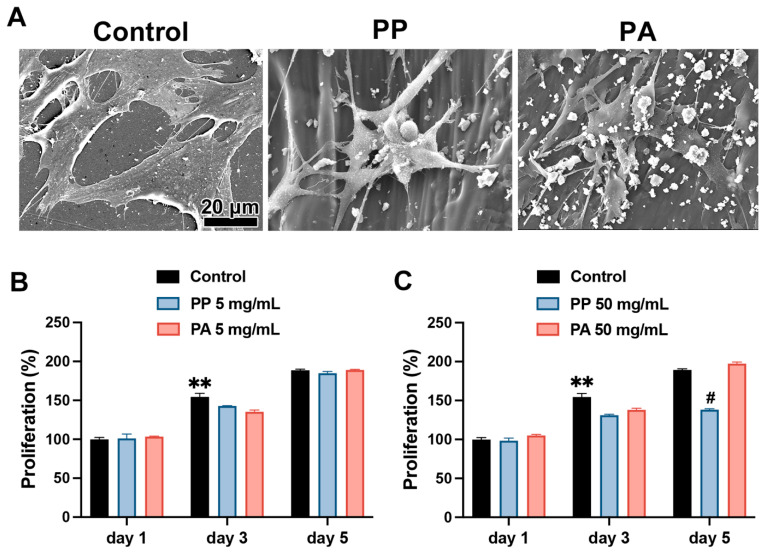
Behaviors of MC3T3E-1 cells cultured with PP and PA materials. (**A**) SEM images of MC3T3E-1 cells cultured on disks of Control cell culture plastic (Control), PP and PA. Scale bar applies to all images. (**B**) Cell proliferation in the tested groups at 1, 3 and 5 days post-seeding with material eluates at 5 mg/mL. (**C**) Cell proliferation in the tested groups at 1, 3 and 5 days post-seeding with material eluates at 50 mg/mL. # denotes significantly lower values than all the other modalities (*p* < 0.05), and ** denotes values significantly higher than those of all the other treatment modalities (*p* < 0.05).

Furthermore, MC3T3E-1 cells were cultured on PP and PA disks. The cells are spread and became elongated when cultured on Control culture plastic slips (Figure 3A). The PP and PA allowed cells to stretch and attach; nevertheless, fewer attached cells were observed on PP and PA when compared to Control (Figure 3A). The attachment behavior on PP and PA disks was similar (Figure 3A). The results of the cell proliferation assay demonstrated that both PP and PA eluates significantly inhibited cell proliferation on day 3 at the tested concentrations (Control vs. PP, *p* = 0.0162; Control vs. PA, *p* < 0.0001 at 5 mg/mL; Control vs. PP, *p* < 0.0001; Control vs. PA, *p* < 0.0001 at 50 mg/mL; Figure 3B,C). However, a lower concentration of the PP and PA eluates (5 mg/mL) did not affect cell proliferation at 5 days post-seeding (Figure 3B). However, cell proliferation was inhibited at 5 days post-seeding with 50 mg/mL PP eluate compared with that after Control and PA treatment (Control vs. PP, *p* < 0.0001; PP vs. PA, *p* < 0.0001; Figure 3C).

### 3.2. In Vivo Assessment of the 3D-Printed Cutting Guide Materials During Bone Healing

PP and PA shavings were implanted in the bone defects along with autologous bone grafts and the effects of the additives PP and PA on bone formation were evaluated in a rat calvarial bone defect model. The bone defects after 2 and 8 weeks of healing was first observed by micro-CT.

**Figure 4:**
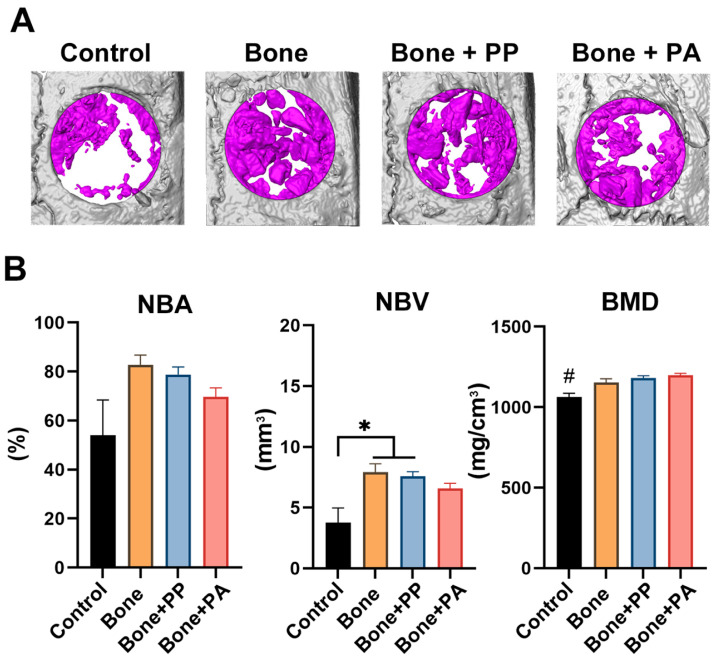
Micro-CT analysis of 4 groups: (1) defect only (Control), (2) autograft (Bone), (3) autograft + PP shavings (Bone + PP), and (4) autograft + PA shavings (Bone + PA) at 2 weeks post-implantation. (**A**) Representative 3D-reconstructed images of each sample. The violet color indicates new formed bone in the defect (volume of interest; VOI). (**B**) Quantitative analysis of the micro-CT images, including the new bone area from the top view (NBA, %), new bone volume (NBV, mm^3^), and bone mineral density (BMD, mg/cm^3^), at 2 weeks post-surgery (n = 5). # denotes significantly lower values than all the other modalities (*p* < 0.05), and * indicates statistically significant differences between groups (*p* < 0.05).

**Figure 5:**
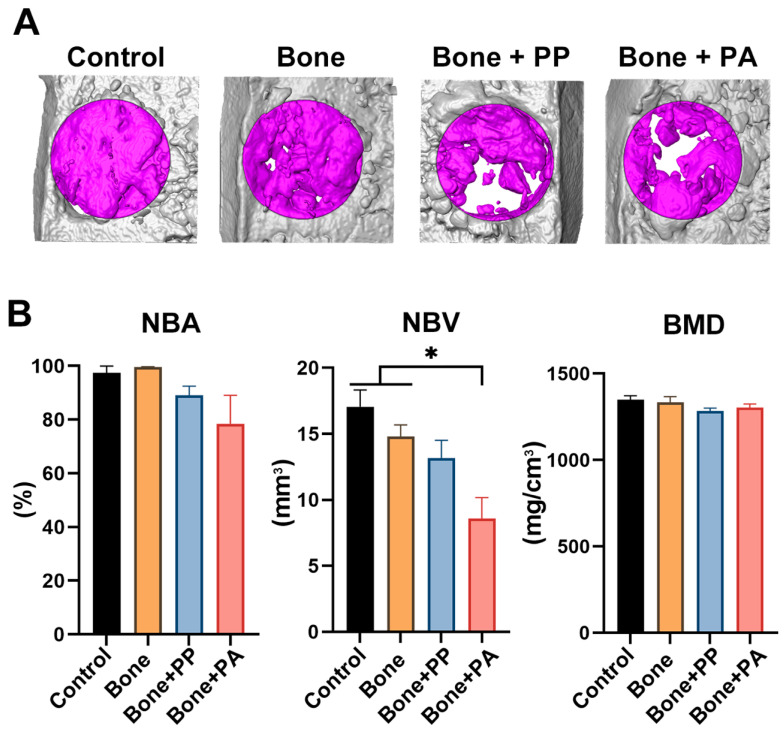
Micro-CT analysis of 4 groups: (1) defect only (Control), (2) autograft (Bone), (3) autograft + PP shavings (Bone + PP), and (4) autograft + PA shavings (Bone + PA) at 8 weeks post-implantation. (**A**) Representative 3D-reconstructed images of each sample. The violet color indicates new bone in the bone defect (VOI). (**B**) Quantitative analysis of the micro-CT images, including NBA (%), NBV (mm^3^), and BMD (mg/cm^3^), at 8 weeks post-surgery (n = 5). # denotes significantly lower values than all the other modalities (*p* < 0.05), and * indicates statistically significant differences between groups (*p* < 0.05).

At 2 weeks, the Control group (defect only) showed bone healing, with approximately 50% new bone covering the defect. Owing to autologous bone grafting, compared with the Control group, the three test groups, namely, the Bone, Bone + PP, and Bone + PA groups, presented increased new bone volume and bone mineral density (Figure 4). A lower BMD was observed in the Control group than in the other groups after 2 weeks (Figure 4B). At 8 weeks, approximately 100% bone healing was observed in the Control group (Figure 5). The extent of bone healing in the Bone and Control groups were similar. However, compared with Control and bone grafting alone (Bone group), PP and PA inhibited bone healing (lower new bone volume/area) at 8 weeks, although the difference was not statistically significant (Figure 5). In particular, the NBV showed significantly lower in the PA group than in the Control and Bone groups (Figure 5B). There were no differences in BMD at 8 weeks among the groups (Figure 5B).

**Figure 6:**
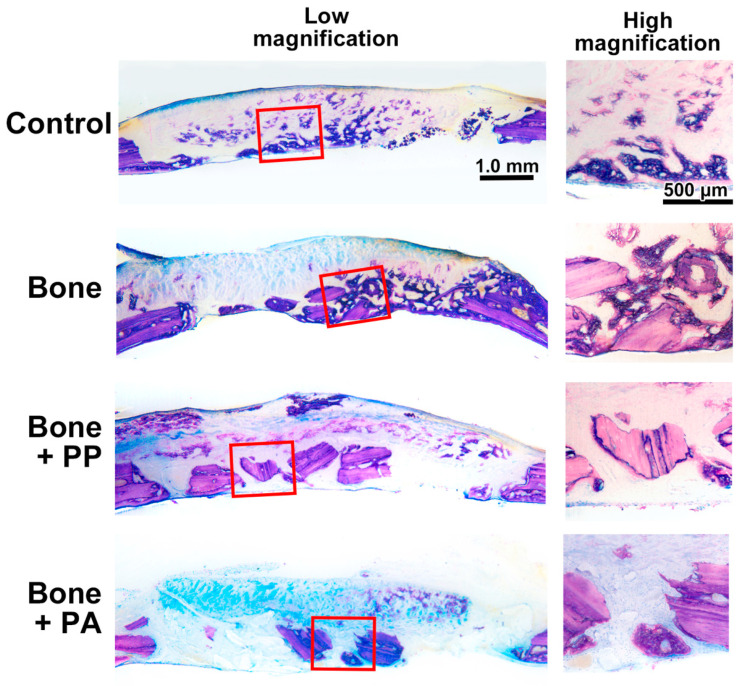
Representative images of bone defects at 2 weeks in the Control (defect only), Bone, Bone + PP, and Bone + PA groups. The left panels present sagittal overviews of the defect stained with toluidine blue and fuchsin, while the right panels display magnified views of the lateral regions of the initial bone defects highlighted by squares in the left panels. Scale bar applies to all images in each magnification.

**Figure 7:**
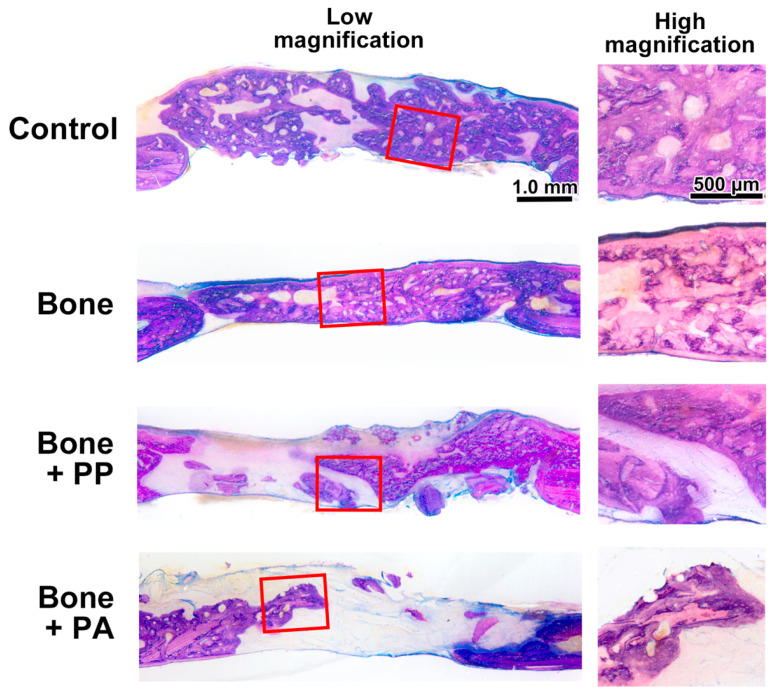
Representative images of bone defects at 8 weeks in the Control (defect only), Bone, Bone + PP, and Bone + PA groups. The left panels present sagittal overviews of the defect stained with toluidine blue and fuchsin, while the right panels display magnified views of the lateral regions of the initial bone defects highlighted by squares in the left panels. Scale bar applies to all images in each magnification.

Thereafter, undecalcified ground sections stained with toluidine blue and fuchsin were subjected to quantitative histomorphometric analysis. All the samples did not show any signs of infection induced by the implanted materials at both 2 and 8 weeks (Figure 6 and Figure 7). Additionally, the PP and PA particles appeared as clear slits that were not stained by toluidine blue or fuchsin (Figure 6 and Figure 7).

**Figure 8:**
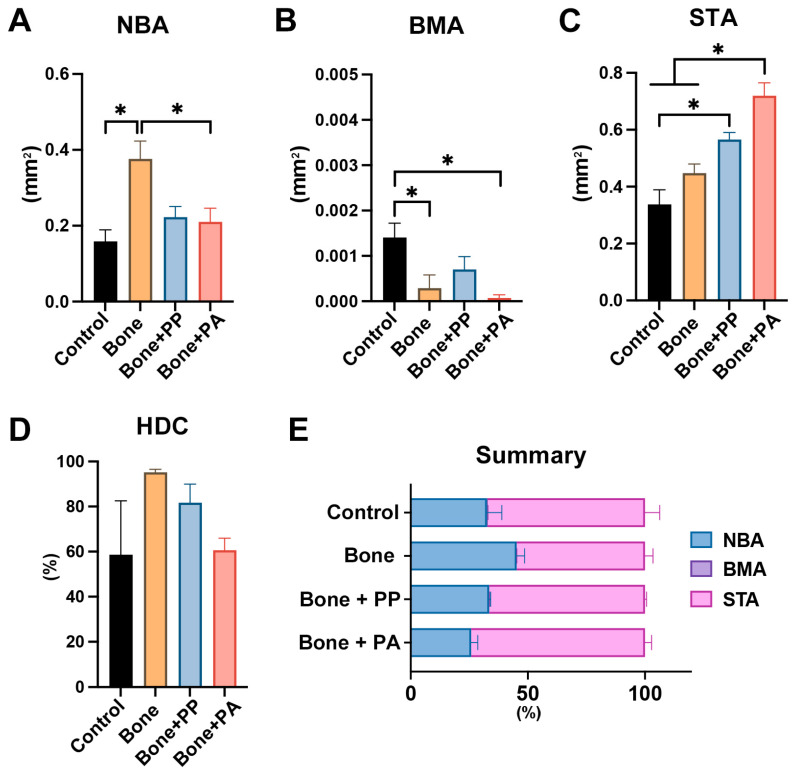
Histomorphometric evaluation of area parameters at 2 weeks post-surgery: (**A**) new bone area (NBA, mm^2^), (**B**) bone marrow area (BMA, mm^2^), (**C**) soft/connective tissue area (STA, mm^2^), and (**D**) horizontal defect closure (HDC, %). (**E**) Overview of the area parameters within the region of interest (ROI) (n = 5). * indicates statistically significant differences between groups (*p* < 0.05).

**Figure 9:**
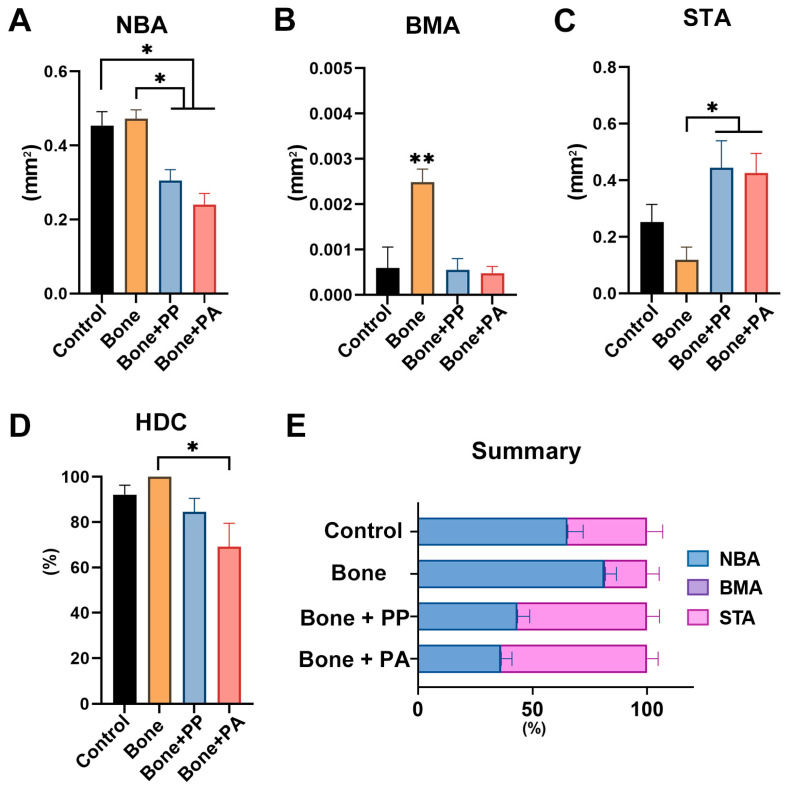
Histomorphometric evaluation of area parameters at 8 weeks post-surgery: (**A**) new bone area (NBA, mm^2^), (**B**) bone marrow area (BMA, mm^2^), (**C**) soft/connective tissue area (STA, mm^2^), and (**D**) horizontal defect closure (HDC, %). (**E**) Overview of the area parameters within the region of interest (ROI) (n = 5). * indicates statistically significant differences between groups (*p* < 0.05), and ** denotes values significantly higher than those of all the other treatment modalities (*p* < 0.05).

Histomorphometric analysis was applied to quantify NBA, BMA, STA, and HDC in the 5-mm defect ROIs at 2 and 8 weeks post-implantation (Figure 8 and Figure 9). The NBA in the Bone group was significantly higher than that in the Control and Bone + PA groups at 2 weeks post-implantation (Figure 8A). Similarly, the greatest HDC was noted in the Bone group at this time point (Figure 8D). However, the BMA in the Control group was larger than those in the Bone and Bone + PA groups at 2 weeks post-implantation (Figure 8B). Additionally, the STA generally showed greater in the Bone + PP and Bone + PA groups than in the other groups (Figure 8C). Overall, the Bone group exhibited a larger greater NBA at 2 weeks because of the bone grafting materials used (Figure 8E). At 8 weeks post-implantation, all groups showed increased bone formation when compared to that at 2 weeks (Figure 8 and Figure 9). The Bone group exhibited greater NBA, BMA and HDC at 8 weeks post-implantation. Additionally, the NBA was smaller, and the STA was larger in the Bone + PP and Bone + PA groups than those in the Bone group (Figure 9A,C). There were no significant differences in any of the measured parameters, including NBA, BMA, STA and HDC, between the Bone + PP and Bone + PA groups at 8 weeks post-implantation (Figure 9A–D). Overall, the NBA was higher and the STA was lower in the Bone group, while these values were lower and higher, respectively, in the Bone + PP and Bone + PA groups at 8 weeks post-implantation (Figure 9E).

## 4. Discussion

3D printing technology is widely applied in the field of oral and maxillofacial surgery [18]. Cutting guides have become valuable and widely used digital tools that improve surgical accuracy and reduce operator-dependent errors and operative times [3,19]. 3D-printed cutting guides are classified as non-implantable devices that contact bone tissues temporally [18]. Patient-specific cutting guides are digitally designed and 3D-printed from materials such as photopolymer resin (PP) and polyamide resin (PA). The PP used in the present study was an in-house system as previously developed and reported [20,21]. In contrast, the PA guide was a widely used system in oral and maxillofacial surgery, designed and manufactured by a third party following online consultation sessions between engineers and surgeons [22]. The main disadvantage with this technique has been the cost and production time, particularly when third-party involvement and engineering support are required.

Many studies have focused on and evaluated the accuracy and clinical outcomes of guides, but few have systematically examined the biological impact of debris from 3D-printed materials [3,11,19]. Nevertheless, the biological impact of their debris generated during surgery remained unclear. The fine resin particles generated intraoperatively may enter bone defects or the surrounding soft tissues via surgical instruments, raising concerns that they may adversely affect bone regeneration and the healing process. The chemical analyses of PP and PA shavings were not conducted in the present study, as the tested materials are already commercially available and are considered non-toxic when used according to the manufacturers’ recommended protocols. Moreover, eluates in water-soluble cell culture media from PP and PA shavings were regarded as minimally toxic. Instead, we focused on examining the biological effects of material shavings generated through clinically relevant methods.

Ultrasonic cutting devices enable minimally invasive, precise bone cuts while reducing the risk of vascular and nerve damage because of their selective cutting ability and microvibration technology [23]. Lohn et al. evaluated the accuracy of various slot properties in 3D-printed cutting guides for free fibular flap elevation utilizing both saws and ultrasonic cutting devices [24]. Their findings indicated that piezosurgery, in which an ultrasonic device is used, provided marginally greater accuracy owing to its slower operation, which enables enhanced visual control, whereas both piezosurgery and conventional saws produced similar clinical outcomes in segmental osteotomy and U-shaped mandibular reconstruction [24]. In the present study, damage to the PP and PA material surface caused by two different cutting devices, namely, a conventional reciprocating saw and an ultrasonic device, was observed by SEM (Figure 1). Surface damage did not seem to influence the accuracy of the jaw resection itself. However, the surface on both tested materials (PP and PA), were damaged by both cutting devices. Furthermore, compared with PA shavings, PP shavings were generally smaller and sharper, as shown in Figure 2.

In cell culture experiments, no significant differences were observed in cell viability among the Control, PP, and PA groups on day 1, suggesting that the effects of the material shavings were minimal for up to 24 h (Figure 3). By day 3, cell proliferation was significantly impaired in both the PP and PA groups when compared with that in the Control group at the tested concentrations of material eluates. On day 5, compared with the Control and PA treatments, the higher concentration of PP eluates (50 mg/mL) resulted in significantly lower cell viability (Figure 3C). Most photosensitive resin materials are known cytotoxic because of the unreacted double bond in their structure and residual photoinitiator in the material [25]. SEM images revealed differences in shaving size between PP and PA, which may influence cellular toxicity. Resin-based composites generally exhibit dose- and time-dependent cytotoxicity, with higher concentrations and longer exposure durations leading to increased toxic effects [26]. The concentrations used in the present study might not be considered clinically relevant but in fact be excessively high. In vitro findings should be interpreted with caution because monomer levels are likely lower in vivo and vary depending on surgical conditions [26].

A rat calvarial bone defect model was selected for the present in vivo study to further evaluate the biological effect of PP and PA during bone healing, since it is a common and simple model that allows direct observation of bone formation [27]. Defects 5 mm in diameter are indeed not critical in size, as we detected complete healing at 8 weeks after surgery in the Control (defect only, empty) group (Figure 5 and Figure 7). However, in clinical practice, following segmental mandibulectomy, bone reconstruction—such as fibular or iliac bone grafting—is typically performed in parallel. In these situations, shavings from the cutting guide may affect the bone–bone contact regions between the residual and reconstructed segments. Therefore, the selected rat bone defect model might provide a reasonable framework to evaluate bone healing under contamination with shavings derived from cutting guide materials.

The bone healing process in the groups was observed by micro-CT and histological analysis at 2 and 8 weeks post-surgery. Interestingly, compared to the Bone group, the PA shavings resulted in less bone formation in the defects at 8 weeks (Figure 5 and Figure 8). Nevertheless, no significant differences were found in the NBA or NBV between the Bone + PP and Bone + PA groups (Figure 5 and Figure 8). The PP and PA shavings remained in the defects for long periods, are resistant to degradation or absorption, and might induce local chronic inflammation and foreign body giant cell reactions. Unfortunately, however, undecalcified ground sections cannot be used to histologically observe foreign bodies or inflammatory cells because of the limitation of section thickness. The resin shavings in the sampled tissues could not be decalcified using conventional acid or EDTA solutions; therefore, undecalcified sections were prepared in the present study. Detailed histological analyses—such as fibrotic capsule formation, inflammatory infiltrates, osteoclastic activity, and cellular interactions with debris—will be important points for future investigation.

Furthermore, the rat calvarial bone defect model does not completely mimic the complex environment of the human jaw and the bone remodeling process in the long term. Future studies should include molecular biological evaluations of inflammatory markers and bone formation-related genes and validate these findings in large animal models and clinical studies. Nevertheless, the outcomes of the present study provide new insights into concerns regarding 3D-printed cutting guide materials that have not been reported previously. Both the PP and the PA used in the present study are ISO-approved materials for medical use as cutting guides that can be attached to the body directly for up to 24 h. However, shavings or debris from the PP and PA generated during the jaw cutting procedure may remain in the body for more than 24 h. Surgeons should be aware of the potential risks associated with the prolonged presence of foreign materials during and after surgery.

## 5. Conclusions

In vitro and in vivo experiments demonstrated that the evaluated 3D-printed cutting guide resin materials, PP and PA, are biocompatible when used according to the manufacturer’s protocol. However, compared with the Control plastic, the shavings derived from both the PP and the PA were found to be more cytotoxic and impaired bone healing in a rat experimental model. Surgeons should be aware that polymer shavings generated during jaw cutting with 3D-printed guides may adversely affect bone healing because of the prolonged presence of material debris within the body.

## Data Availability

The original contributions presented in this study are included in the article. Further inquiries can be directed to the corresponding author.

## Abbreviations

The following abbreviations are used in this manuscript:

PP	Photopolymer resin
PA	Polyamide resin
CAD/CAM	Computer-aided design/manufacturing
3D	Three-dimensional
CAS	Computer-aided surgery
VPP	Vat photopolymerization
PBF	Powder bed fusion
SLA	Stereolithography
SLS	Selective laser sintering
SEM	Scanning electron microscopy
FBS	Fetal bovine serum
NBA	New bone area
NBV	New bone volume
BMD	Bone mineral density
BMA	Bone marrow area
STA	Soft/connective tissue area
HDC	Horizontal defect closure
VOI	Volume of interest
ROI	Region of interest
